# Supplementary material for: Basic Affective Systems and Sex Differences in the Relationship between Anger and Fear
Source: Int J Environ Res Public Health. 2024 Sep 24;21(10):1266. doi: 10.3390/ijerph21101266 (PMC11507658; doi:10.3390/ijerph21101266)
Supplement: Supplementary file 1 [file ijerph-21-01266-s001.zip › ijerph-3172664-supplementary.pdf]

Table S1. Cronbach's alpha, mean and standard deviation of each basic affective system, showing the items and the additional scores that make up each affective system in ANPS 3.1.

	Cronbach's alpha	Mean±SD
SEEKING		
anps1+anps17+anps33+anps49+anps65+anps81+anps97-anps9-anps25-anps41-anps57-anps73-anps89-anps105+35	,7067	45,67±6,86
FEAR		
anps2+ anps18+ anps34+ anps50+ anps66+ anps82+ anps98- anps10-anps26-anps42- anps58- anps74- anps90-anps106+35	,8719	37,31±9,69
CARE		
anps3+ anps19+ anps35+ anps51+ anps67+ anps83+ anps99- anps11 -anps27 -anps43- anps59 -anps75- anps91- anps107+35	,7565	44,36±7,96
ANGER		
anps4+anps20+anps36+anps52+anps68+anps84+anps100-anps12-anps28-anps44-anps60-anps76-anps92-anps108+35	,8197	29,48±8,91
PLAY		
anps5+anps21+anps37+anps53+anps69+anps85+anps101-anps13-anps29-anps45-anps61-anps77-anps93-anps109+35	,8246	38,54±8,35
PANIC		
anps6+anps22+anps38+anps54+anps70+anps86+anps102-anps14-anps30-anps46-anps62-anps78-anps94-anps110+35	,784	36,61±8,02

Table S2. Covariance analysis, considering the various basic affective systems in relation to sex and age.

	Covariance model		Interaction model
	sex	age	sex*age
FEAR	,459	<,001	,577
SEEK	,386	,456	,658
CARE	<,001	,926	,100
ANGER	,784	,003	,651
PLAY	,228	,001	,453
SADNESS	<,001	,176	,855
Social Anxiety	,004	,225	,381
Dominace	,001	,019	,162
